# Dental health status and oral health behaviours of patients with facial burn in Pakistan

**DOI:** 10.1186/s12903-019-0819-0

**Published:** 2019-06-26

**Authors:** Farooq Ahmad Chaudhary, Basaruddin Ahmad, Ulfat Bashir

**Affiliations:** 1 Dental College, HITEC Institute of Medical Sciences, Taxila, Pakistan; 2 School of Dental Sciences, Universiti Sains Malaysia, Kubang Kerian, Malaysia; 3 Islamic International Dental College, Riphah International University, Islamabad, Pakistan

**Keywords:** Burn, Oral health, Behaviours, Psychological, DMFT, CPI, OHI-S, Self-perceived

## Abstract

**Background:**

There is a limited understanding about the oral health of patients with facial burn, hence the aim was to describe the oral health status and the related risks factors.

**Methods:**

This cross-sectional study had randomly and systematically recruited facial burn patients from the Burn Care Center, Pakistan Institute of Medical Sciences, Islamabad, from June of 2016 to July of 2017. Intraoral examination recorded the DMFT, CPI and OHI-S. Information on the socio-demographic status, self-perceived oral health, oral health behaviours were collected using a self-administered questionnaire and; the burn characteristics were obtained from the patients’ medical record. The t-test, ANOVA, SLR, and chi-square test were used to examine the relationship between oral health and each factor. A parameter was derived from the clinical indices using the principal component analysis and used in the multiple linear regression analysis to determine the important factors associated with oral health status.

**Results:**

A total of 271 burn patients (69% female and 31% male) had participated in the study. All of the participants had caries with mean DMFT = 10.96 (95%CI: 10.67, 11.25). There were 59.0% (95%CI: 53.15, 64.93%) and 66.1% (95%CI: 60.38, 71.73%) of the participants who had periodontitis and poor oral hygiene respectively. About 79 and 80% of the participants rated their dental and periodontal status as poor. About 78% reported brushing once daily and 89% did not practice regular dental visit. The DMFT, CPI and OHI-S were associated with the burn characteristics and oral health behaviours (*p* < 0.05). Dental anxiety, cost and social issues were the most cited reasons for not utilising oral health services. Greater burn severity, the longer time elapsed since the burn incident, and dental anxiety were associated with poorer oral health status and; brushing twice or more and regular dental visit, with better status (*p* < 0.01).

**Conclusion:**

Patients with oro-facial burn injury had a generally poor oral health and, the risks are greater in those with a more severe and wider area of injury, the longer time elapsed since the burn incident and dental anxiety; but a good oral hygiene practice and regular dental visits were protective against the risk.

## Background

Burn injuries may have devastating impacts on a victim and is one of the major public health problems in the developing world [1, 2]. A burn to the oro-facial region may have compounding impacts that include physical changes to the oral-motor structure, morphology, mobility and oral functions. Oral burn contracture, a condition where the skin tightens as a result of scarring, may lead to microstomia, which can affect the ability to perform daily activities such as mastication, swallowing and speech [3–6]. Other complications include the loss of facial and labial sensation, inadequate oral competence which cause chronic drooling and, difficult access for oral hygiene care or dental treatment [7]. The effects of oro-facial changes were not limited to the physical aspects but also on the psycho-social quality of life as it impacts growth, eating, speaking, self-esteem, personal relationships, financial, social interactions and social well-being [8, 9].

Technological advancement in burn care techniques to improve oral health related quality of life such as corrective surgery, implants, and complex prostheses; in tandem with psychological rehabilitation, could improve the survival rate and coping with the impact of the burn incident, particularly in the severely burned cases [10]. These, however, benefit the victims in the developed countries more than those in the other parts of the world because of the availability of services and cost. Nevertheless, the demand and expectation for a better care of burn victims and to improve the general and oral health related quality of life is likely to continue to increase and hopefully it would include the less affluent groups in the future [11, 12]. Because of that, it is essential to understand the burden of oral health problems in the oro-facial burn community in order to plan the much-needed rehabilitation and treatment services. However, there is currently little description of the oral health burden of that community in the literature, hence the aim to describe the oral health problems and the factors that influence their oral health condition.

## Methods

This cross-sectional study was carried out at the Burn Care Center, Pakistan Institute of Medical Sciences in Islamabad, Pakistan after the ethical approval and study protocol was approved (Reference no. F.1–1/2015/ERB/SZABMU). Burn patients who were visiting the centre for follow-up were included in the study if they were over 15 years-old, sustained the injury over one year, had a greater than 10% total body surface area (TBSA) affected by the burn which involved the face and neck region and, able feed exclusively by mouth [13]. They were systematically and randomly recruited during their follow-up visits at the centre. Sample size was calculated at *N* = 270 based on the prevalence of caries (61.4%) and periodontal disease (34.5%) in the region at 95% confidence level and 10% precision [14, 15].

Consented patients were subjected to an intraoral examination and self-administered questionnaires and, assisted if required. One investigator had carried out all the examination in a room with the patient sitting on a reclined chair and under adequate lighting. Mouth mirror, explorer and periodontal probe were used and, the DMFT, Community Periodontal Index (CPI) (score: 0, 1, 2, 3, 4) and Oral Hygiene Index Simplified (OHI-S) (good, fair, poor) were recorded based on previously described methods [16, 17]. The background information collected were age group, sex, education, employment and, personal and family income. The response to two questions on the self-perceived oral health status, *“What is your opinion regarding the health status of your teeth?”* and *“What is your opinion regarding the health status of your gums?”* were re-classified as “good” (initially recorded as good and very good) and “poor” (not good and bad) [18–20]. Oral health behaviours were assessed by asking about the frequency of tooth brushing (once, twice or more) and whether they had a regular dental check-up in the past year [21]. Barriers to utilization of oral health care was assessed using an open-ended question *“Are there anything, such as cost, anxiety, place, illness, or other problems, that have kept you from going to the dentist?”* and only the first response from the patients’ own words/phrases were applied in the analysis [22, 23]. Because the responses varied between participants and for simplicity of interpretation, the meaning of the responses were classified under 5 themes: cost, dental anxiety, social issues, distance and self-perceived. The words/phrases such as ‘*expensive’, ‘cannot afford’* or *‘lack of income’* was represented by the *cost*, which reflected the high cost of treatment. The *dental anxiety* represented the ‘*shyness because of dental condition’, ‘nervousness’, ‘fear of dental treatment’* or *‘using spiritual healing’*; which reflected the participants’ fear of dental treatment or sought alternative treatment to avoid a dentist. The *social issues* represented the ‘*stigma’, ‘feeling embarrassed’* and *‘dependent on others for financial’* or *‘logistic support’* to reflect the participants’ feeling and esteem when they faced other people or, relied on favours from others for their needs. Responses such as ‘*no transportation’* and *‘living far from clinics’* were classified as the *distance*; and ‘*health is not important’* or *‘no treatment needed’,* as the *self-perceived* belief that the participants did not need any dental treatment. Three burn characteristics information were obtained from the medical record; the degree of burn which described the severity of injury based on the depth of skin burned [24], the total body surface area (TBSA) affected by the injury [25] and, the time elapsed after the burn incidence.

### Statistical analysis

Summary statistics were obtained for all the variables. The t-test, ANOVA and simple linear regression (to examine the increasing trend effect) were used to examine the relationships between DMFT and the risk factors and; the chi-square test, for the CPI and OHI-S. To identify the factors associated with an overall oral health status and because the DMFT, CPI and OHI-S indices were correlated, a new parameter was derived by converting them into a single parameter *clinical oral status* using the component factor analysis. The procedure was applied with no rotation and one factor (mean = 0.0, sd = 1, min = − 3.17, max = 1.91) with Eigenvalue = 2.5 that explained 82.8% of the variance was extracted and saved. An increasing value of the parameter indicated a worse clinical oral status. It was then used as an outcome variable in the multiple linear regression analysis to identify the factors that influence oral health in burn patients using the stepwise method with p_inclusion_ = 0.05, and p_removal_ ≥ 0.1. All analysis was performed at 5% significance level and carried out in IBM SPSS software version 22.0.

## Results

A total of 300 subjects were approached and, only 271 (90.3% response rate) had consented and completed the oral examination and questionnaires. The majority of the sample were females, between 15 and 34 years-old, had less than 12 years of schooling, unemployed and from the low-income background (less than 24,000 PKR for both personal and family income) (Table 1). There were slightly greater percentages of participants who had the second degree burn (52%), 10–20% TBSA (54%) and injuries that were sustained more than 3 years (54%). Fire\flames was the major cause of burn injuries in the sample (41%).

**Table 1:** Sociodemographic characteristics of the participants (*N* = 271)

Characteristics	Number (%)
Age	
15–24	89 (32.8)
25–34	125 (46.1)
35–44	40 (14.8)
45+	17 (6.3)
Gender	
Male	85 (31.4)
Female	186 (68.6)
Education level	
0–5 years of schooling	73 (26.9)
6–12 years of schooling	176 (64.9)
13+ years of schooling	22 (8.1)
Employment status	
Full time job	49 (18.1)
Part time job	76 (28.0)
Unemployed	133 (49.1)
Others (student, retired)	13 (4.8)
Personal income (PKR)	
5000–14,000	178 (65.7)
15,000–24,000	47 (17.3)
25,000–34,000	24 (8.9)
35,000 +	22 (8.1)
Family income (PKR)	
15,000–24,000	139 (51.3)
25,000–34,000	59 (21.8)
35,000 +	73 (26.9)
Degree of burn injury	
Second degree burn	141 (52.0)
Third degree burn	130 (48.0)
Total body surface area (TBSA)	
10–20% TBSA	146 (53.9)
More than 20% TBSA	125 (46.1)
Time of incident	
1–2 years	47 (17.3)
2–3 years	78 (28.8)
3–4 years	105 (38.7)
4+ years	41 (15.1)
Causes of burn injury	
Fire\Flame	112 (41.3)
Scald\Stream	77 (28.4)
Chemical\assault	58 (21.4)
Electrical\others	24 (8.8)
DMFT, mean (SD)	11.0 (2.4)
CPI	
Bleeding	31 (11.4)
Calculus	80 (29.5)
4-5 mm pockets	117 (43.2)
6 mm and more pockets	43 (15.9)
OHI-S	
Good	7 (2.6)
Fair	85 (31.4)
Poor	179 (66.1)
Self-perceived dental health	
Good	58 (21.4)
Poor	213 (78.6)
Self-perceived periodontal health	
Good	55 (20.3)
Poor	216 (79.7)
Daily frequency of toothbrushing	
One time	212 (78.2)
Two times	59 (21.8)
Regular check-up	
Yes	29 (10.7)
No	242 (89.3)
Barriers to utilization of oral health care services	
Cost	68 (25.1)
Distance	25 (9.2)
Psychological	125 (46.1)
Social	42 (15.5)
Self-perceived	11 (4.1)

All of the participants had caries and the mean DMFT was 10.96 (sd = 2.41) (Table 1). About 59% of the participants had periodontal pocket greater than 4 mm in at least one site and 66% had poor oral hygiene. In contrast to the clinical indices, the percentage of the participants who perceived their dental (79%) and periodontal (80%) health as poor was greater. The majority had practised tooth brushing once a day (78%) and did not visit a dentist in the past year for a regular checkup (89%). Forty-six percent of the participants cited dental anxiety as the primary reason for not visiting a dentist, followed by cost (25%) and social issues (16%).

The results in Table 2 showed that poorer caries severity periodontal and oral hygiene statuses were significantly associated with the burn characteristics, oral health behaviours and age (*p* < 0.001). The participants with a more severe degree of burn and TBSA, and the longer time elapsed since the burn incident, practiced tooth brushing once a day and did not visit a dentist in the past year had greater mean DMFT, CPI > 2 and poor OHI-S score; compared to the corresponding counterparts. Older participants had poorer caries severity and, periodontal and oral hygiene statuses. Caries severity was poorer in males than females. The perceived dental and periodontal status was not associated with any of the factors. The participants who cited dental anxiety as the primary barrier to the utilization of oral health care services had the greatest mean DMFT and a greater percentage of them had periodontal pockets and poor oral hygiene statuses.

**Table 2:** The summary of the DMFT, CPI and OHI-S indices, and self-perceived dental and periodontal status by the burn characteristics, social demographic and oral health behaviours in burn patients (N = 271)

	DMFT Mean (SD)	CPI n(%)				OHI-S n(%)			Self-perceived dental status n(%)		Self-perceived periodontal status n(%)	
		1	2	3	4							
		Bleeding	Calculus	4-5 mm pockets	> 6 mm pockets	Good	Fair	Poor	Good	Bad	Good	Bad
Degree of burn												
2nd degree	9.4 (2.1)	31 (21.9)	63 (44.6)	42 (29.7)	5 (3.5)	7 (4.9)	76 (53.9)	58 (41.1)	25 (17.7)	116 (82.2)	24 (17.0)	117 (83.0)
3rd degree	12.6 (1.4)	0	17 (13.0)	75 (57.6)	38 (29.2)	0	9 (6.9)	121 (93.0)	33 (25.3)	97 (74.6)	31 (23.8)	99 (76.2)
*p*-value	< 0.001	< 0.001				< 0.001			0.08		0.3	
TBSA												
10–20%	9.5 (2.2)	31 (21.2)	63 (43.1)	45 (30.8)	7 (4.7)	7 (4.7)	77 (52.7)	62 (42.4)	29 (19.8)	117 (80.1)	28 (19.1)	118 (80.8)
More than 20%	12.5 (1.4)	0	17 (13.6)	72 (57.6)	36 (28.8)	0	8 (6.4)	117 (93.6)	29 (23.2)	96 (76.8)	27 (21.6)	98 (78.4)
*p*-value	< 0.001	< 0.001				< 0.001			0.3		0.3	
Time elapsed												
12–24 months	7.0 (1.7)	30 (63.8)	17 (36.1)	0	0	7 (14.8)	39 (82.9)	1 (2.1)	8 (17.1)	39 (82.9)	8 (17.1)	39 (82.9)
25–36 months	10.4 (0.9)	1 (1.3)	44 (56.4)	28 (35.8)	5 (6.4)	0	36 (46.1)	42 (53.8)	17 (21.7)	61 (78.2)	16 (20.5)	62 (79.4)
37–48 months	11.9 (0.9)	0	19 (18.0)	72 (68.5)	14 (13.3)	0	10 (9.5)	95 (90.4)	26 (24.7)	79 (75.2)	24 (22.8)	81 (77.1)
48 + months	13.8 (1.4)	0	0	17 (41.4)	24 (58.5)	0	0	41 (100.0)	7 (17.1)	34 (82.9)	7 (17.0)	34 (83.0)
*p*-value	< 0.001^1^	< 0.001				< 0.001			0.6		0.8	
Causes of burn												
Fire\flame	11.3 (1.9)	5 (4.5)	34 (30.3)	56 (50.0)	17 (15.1)	1 (0.8)	27 (24.1)	84 (75.0)	23 (20.5)	89 (80.5)	20 (17.8)	92 (82.2)
Scald\stream	10.6 (1.8)	7 (9.1)	28 (36.3)	33 (36.3)	9 (11.6)	0	27 (35.0)	50 (65.0)	15 (19.4)	62 (81.6)	16 (20.7)	61 (80.3)
Chemical\assault	11.9 (2.3)	4 (6.9)	13 (22.4)	25 (43.1)	16 (27.5)	0	17 (29.3)	41 (70.6)	16 (27.5)	42 (72.4)	14 (24.1)	44 (76.9)
Electrical\others	7.62 (3.1)	15 (62.5)	5 (20.8)	3 (12.5)	1 (4.1)	6 (2.5)	14 (58.3)	4 (16.6)	4 (16.6)	20 (84.4)	5 (20.8)	19 (80.2)
*p*-value	< 0.001	< 0.001				< 0.001			0.6		0.8	
Age												
15–24	9.9 (2.5)	23 (25.8)	35 (39.3)	25 (28.0)	6 (6.7)	6 (6.7)	37 (41.5)	46 (51.6)	20 (22.4)	69 (77.6)	18 (20.2)	71 (79.7)
25–34	11.8 (1.5)	2 (1.6)	25 (20.0)	71 (56.8)	27 (21.6)	0	22 (17.6)	103 (82.4)	29 (23.2)	96 (76.8)	27 (21.6)	98 (78.4)
35–44	10.1 (2.7)	4 (10.0)	15 (37.5)	14 (35.0)	7 (17.5)	1 (2.5)	17 (42.5)	22 (55.0)	6 (15.0)	34 (85.0)	7 (17.5)	33 (82.5)
45+	11.1 (3.4)	2 (11.7)	5 (29.4)	7 (41.1)	3 (17.6)	0	9 (52.9)	8 (47.0)	3 (17.6)	14 (82.3)	3 (17.6)	14 (82.3)
*p*-value	0.07^1^	< 0.001				< 0.001			0.7		0.9	
Gender												
Male	11.4 (2.2)	4 (4.7)	25 (29.4)	39 (45.8)	17 (20.0)	0	25 (29.4)	60 (70.5)	17 (20.0)	68 (80.0)	17 (20.0)	68 (80.0)
Female	10.7 (2.4)	27 (14.5)	55 (29.5)	78 (41.9)	26 (13.9)	7 (3.7)	60 (32.2)	119 (63.9)	41 (22.0)	145 (78.0)	38 (22.0)	148 (79.5)
*p*-value	< 0.001	0.9				0.2			0.4		0.5	
Education												
0–5	11.1 (2.3)	6 (8.21)	17 (23.2)	34 (46.5)	16 (21.9)	3 (4.1)	19 (26.0)	51 (69.8)	17 (23.2)	56 (76.8)	15 (20.5)	58 (79.4)
6–12	10.7 (2.4)	25 (14.2)	58 (32.9)	70 (39.7)	23 (13.0)	4 (2.3)	62 (35.2)	110 (62.5)	37 (21.0)	139 (79.0)	36 (20.4)	140 (79.5)
13+	11.8 (2.0)	0	5 (22.7)	13 (59.0)	4 (18.1)	0	4 (18.1)	18 (81.8)	4 (18.1)	18 (81.9)	4 (18.1)	18 (81.8)
*p*-value	0.8^1^	0.1				0.2			0.8		0.9	
Employment status												
Full time job	10.5 (2.6)	5 (10.2)	15 (10.2)	22 (30.6)	7 (14.2)	1 (2.0)	18 (36.7)	30 (61.2)	9 (18.3)	40 (81.7)	110 (20.4)	39 (79.6)
Part time job	11.4 (2.2)	6 (7.8)	6 (7.8)	31 (40.7)	16 (21.0)	1 (1.3)	24 (31.5)	51 (67.1)	15 (19.7)	61 (80.3)	15 (19.7)	61 (80.3)
Unemployed	10.8 (2.3)	18 (13.5)	18 (13.5)	61 (45.8)	18 (13.5)	5 (3.7)	39 (29.3)	89 (66.9)	30 (22.5)	103 (77.5)	26 (19.5)	107 (80.4)
Others (student, retired)	10.6 (2.4)	2 (15.3)	2 (15.3)	3 (23.0)	2 (15.3)	0	4 (30.7)	9 (69.2)	4 (30.7)	9 (69.3)	4 (30.7)	9 (69.3)
*p*-value	0.2	0.6				0.8			0.8		0.7	
Personal income												
5000–14,000	10.9 (2.3)	24 (13.4)	48 (26.9)	81 (45.5)	25 (14.0)	5 (2.8)	53 (29.7)	120 (67.4)	40 (22.4)	138 (77.6)	36 (20.2)	142 (79.8)
15,000–24,000	11.7 (2.2)	2 (4.2)	17 (36.1)	17 (36.1)	11 (23.4)	0	15 (31.9)	32 (68.0)	10 (21.2)	37 (78.8)	10 (21.2)	37 (78.8)
25,000–34,000	10.3 (2.9)	3 (12.5)	8 (33.3)	8 (33.3)	5 (20.8)	1 (4.1)	9 (37.5)	14 (58.3)	4 (16.6)	20 (83.3)	5 (20.8)	19 (79.2)
35,000 +	10.5 (3.0)	2 (9.1)	7 (31.8)	11 (50.0)	2 (9.09)	1 (4.5)	8 (36.3)	13 (59.0)	4 (18.1)	18 (81.8)	4 (18.1)	18 (82.9)
*p*-value	0.5^1^	0.4				0.8			0.9		0.9	
Family income												
15,000–24,000	10.9 (2.3)	18 (12.9)	33 (23.7)	66 (47.4)	22 (15.8)	5 (3.5)	35 (25.1)	99 (71.2)	28 (20.1)	111 (79.9)	26 (18.7)	113 (82.3)
25,000–34,000	11.2 (2.3)	4 (6.7)	24 (40.6)	21 (35.5)	10 (16.9)	1 (1.6)	20 (33.8)	38 (64.4)	15 (25.4)	44 (74.6)	14 (31.1)	45 (69.9)
35,000 +	10.7 (2.6)	9 (12.3)	23 (31.5)	30 (41.0)	11 (15.0)	1 (1.3)	30 (41.0)	42 (57.5)	15 (20.5)	58 (79.5)	15 (20.5)	58 (79.5)
*p*-value	0.5^1^	0.3				0.2			0.5		0.4	
Teeth brushing												
Once a day	11.7 (1.7)	6 (2.8)	55 (25.9)	108 (50.9)	43 (20.2)	1 (0.5)	42 (19.8)	169 (79.7)	44 (20.7)	168 (79.2)	41 (19.3)	171 (80.6)
Twice or more	8.23 (2.3)	25 (42.3)	25 (42.3)	9 (15.2)	0	6 (10.1)	43 (72.8)	10 (16.9)	14 (23.7)	45 (77.3)	14 (23.7)	45 (77.3)
*p*-value	< 0.001	< 0.001				< 0.001			0.3		0.2	
Dental checkups												
Yes	7.03 (2.1)	18 (62.0)	10 (34.4)	1 (3.4)	0	4 (13.7)	24 (82.7)	1 (3.4)	6 (20.6)	23 (79.6)	5 (17.2)	24 (83.8)
No	11.4 (1.9)	13 (5.3)	70 (28.9)	116 (47.9)	43 (17.7)	3 (1.2)	61 (25.2)	178 (73.5)	52 (21.4)	190 (79.6)	50 (20.6)	192 (79.4)
*p*-value	< 0.001	< 0.001				< 0.001			0.5		0.4	
Barriers to oral health care												
Cost	10.4 (2.8)	15 (22.0)	20 (29.4)	27 (39.7)	6 (8.8)	4 (5.8)	31 (45.5)	33 (48.5)	17 (25.0)	51 (75.0)	16 (23.5)	52 (77.5)
Distance	10.7 (2.4)	4 (16.0)	10 (40.0)	9 (36.0)	2 (8.0)	0	10 (40.0)	15 (60.0)	7 (28.0)	18 (72.0)	7 (28.0)	18 (72.0)
Dental anxiety	11.6 (1.7)	4 (3.2)	27 (21.6)	66 (52.8)	28 (22.4)	1 (0.8)	22 (17.6)	102 (81.6)	23 (18.4)	102 (82.6)	20 (16.0)	105 (84.0)
Social issues	11.1 (2.2)	4 (9.5)	17 (40.4)	14 (33.3)	7 (16.6)	0	15 (35.7)	27 (64.2)	9 (21.4)	33 (79.6)	10 (23.8)	32 (77.2)
Self-perceived	7.6 (2.2)	4 (36.3)	6 (54.5)	1 (9.1)	0	2 (18.1)	7 (63.6)	2 (18.1)	2 (18.1)	9 (82.9)	2 (18.1)	9 (82.9)
*p*-value	< 0.001	< 0.001				< 0.001			0.7		0.5	

^1^simple linear regression, increasing trend

The multiple linear regression analysis showed that the burn severity, time of incidence age and dental anxiety were statistically and significantly associated with worse clinical oral status and, more frequent brushing and regular dental check-ups were associated with better clinical outcomes in the burn patients (Table 3). Because both of the burn severity and TBSA were strongly correlated (0.86, *p* < 0.001), the latter were excluded from the model presented in Table 3. An alternative model that replaced the burn severity with the TBSA showed a slightly smaller but significant effect (regression coefficient = 0.23, 95%CI: 0.08, 0.38, *p* = 0.002) and very little change to the other covariates.

**Table 3:** Regression coefficient (b) of univariate and multiple linear regressions of the analysis of risk factors for clinical oral status parameter in burn patients (N = 271)

	Univariate analysis b (95%CI)	Regression coefficient for the multiple regression analysis, b (95% CI)		Standardized regression coefficient, b
Degree of burn (Third)	1.29 (1.11, 1.47)^2^	0.30 (0.15, 0.45)	< 0.001	0.15
TBSA (More than 20%)	1.24 (1.05, 1.42)^2^	–	–	–
Time of incident^1^	0.89 (0.82, 0.96)^2^	0.60 (0.51, 0.69)	< 0.001	0.57
Age^1^	0.16 (0.02, 0.30)^2^	0.10 (0.03, 0.16)	0.004	0.08
Teeth brushing (twice)	−1.51 (−1.74, −1.28)^2^	0.29 (−0.47, − 0.11)	0.002	− 0.12
Dental checkups (yes)	−1.81 (−2.13, − 1.49)^2^	0.53 (− 0.75, − 0.31,)	< 0.001	−0.16
Barriers to oral health				
care^3^				
Cost	−0.74 (− 1.01, − 0.47)^2^	–		
Distance	−0.53 (− 0.93, − 0.13)^2^	–		
Dental anxiety	0.35 (0.19, 0.52)^2^	0.21 (0.09, 0.33)	< 0.001	0.11
Social issues	−0.34 (− 0.66, − 0.17)^2^	–		
Self-perceived	−1.67 (−2.24, − 1.11)^2^	–		
Gender (Female)	−0.29 (− 0.55, − 0.04)^2^	–		
Education (trend)^1^	−0.00 (− 0.22, 0.21)	–		
Employment^3^				
Unemployed	−0.03 (− 0.20, 0.138)	–		
Full time	−0.06 (− 0.39, 0.27)			
Part time	−0.09 (− 0.66, 0.49)			
Others (student / retired)	0.17 (−0.11, 0.46)			
Personal income^1^	−0.03 (− 0.16, 0.10)	–		
Family income^1^	−0.07 (− 0.21, 0.07)	–		

^1^examined as increasing trend, ^2^*p* < 0.05, ^3^entered as dummy variables in the multiple regression model

## Discussion

This report is the first to describe the oral health status of burn patients and focussed on severe cases of oro-facial burn from a single burn centre. The greater percentage of second degree burn cases in the sample, which was mostly due to fire and scalding, was consistent with earlier reports [26–28]. The percentage of patients with 10–20% total body surface area (TBSA) burned in the sample was similar to studies in Sweden and the USA [29, 30] but lower compared to other regional studies where the cases with TBSA greater than 20% were more prevalent [25, 31]. However, the variation could be due to the inclusion criteria that the oro-facial is affected [27].

The participants were found to be burdened with caries, periodontal disease and poor oral hygiene. Because there was no earlier report on the oral health status of burn patients in the literature, it was not possible to contrast their status to any population. It is also not clear from this study whether the status had deteriorated after the burn incident. But the DMFT was, however, found to be significantly greater (mean DMFT = 8.02, *p* < 0.01), and the percentage of those with deep periodontal pocket (CPI ≥ 3) was 1.5 times greater (35.5%, *p* < 0.05), than that reported in a previous study in Pakistan [14]. The perceived dental and periodontal statuses reported by the participants were not consistent with the clinical indices. Further examination of the data showed that the participants who rated their dental health as good had overestimated their status where the DMFT (mean = 11.2, sd = 2.02, *n* = 58) was not different from those who rated it as poor (mean = 10.9, sd =2.51, *n* = 213). And similarly, from the 20% of the participants who rated their periodontal health as good, none actually has had a clinically healthy periodontium (CPI = 0). Thus, suggesting that the assessment of oral health status using a perceived measure may not be a reliable indicator for this community.

The analysis found two important factors associated with poor oral health status in burn patients. First is the characteristics of burn injury where the participants with greater severity of burn and amount of burnt skin area, and the longer time elapsed since the burn incident had a worse oral health status. Scarring of the skin, muscles and mucosa due to burn does not only leave an unfavourable physical appearance on the oro-facial area, but it may also distort and limit mouth opening, lead to microstomia and cause pain on forced-opening. During the oral examination of this study, the researcher had noticed that a number of participants were uncomfortable and in pain when they were asked to open their mouth wide and for too long and; because of that a detailed intraoral examination and maxillofacial mobility and function parameters that have been planned earlier were excluded to minimise assessment time and discomfort. The similar pain could limit access to the oral cavity and makes oral hygiene practice uncomfortable and inefficient. Patients with an extended burn injury to the upper limbs, hands and fingers could have a greater disadvantage to carry out oral hygiene care effectively and could partly explain the lower frequency of brushing in the sample [32, 33]. It is also plausible, in really severe burn cases where the salivary glands are affected, that the salivary flow is reduced and the longer time elapsed after the burn incident only increases the period of exposure of the teeth and oral structures to plaque and the risk of developing caries [34]. Changes to facial appearance could also impact on self-esteem and depression and; these have been reported to affect health behavior [35]. These, however, are not captured in the present study.

The second factor associated with poor oral health status is the oral health behaviours of burn patients. The analysis suggested that good oral hygiene practices, as simple as brushing twice or more per day, and regular dental visits have a protective effect on burn patients, consistent previous studies [36, 37]. However, the majority of the participants in this study did not visit a dentist in the past year despite their poor oral health condition and the most likely explanation is dental anxiety. When asked about the reason for not utilizing the dental health services, majority of the participants cited dental anxiety as the primary barrier; and they also had the worst oral health condition compared to those who cited other reasons. Previous studies had reported associations between dental anxiety and depression and, lower frequency of dental visit [38–40]. Being outside the house will expose the facial burn patients to the public and less familiar social environment, and in the context of the local society, this could make them feel uncomfortable, anxious, embarrassed and depressed and, hence, avoid travelling to the dentist [41–44]. The cost of dental care is another barrier and was a concern to those from the lower socioeconomic background. The high cost of dental treatment could be an additional burden to the patients and their families on top of the expenses spent on treatment and hospitalization costs for the injury [45, 46]. Because of these reasons, the burn patients is missing out the professional help that could improve their knowledge, awareness and skill to take care of their oral health.

It can be deduced from this study that oral health behaviours are important determinants of the oral health status of burn patients in Pakistan. To overcome the issue, in tandem with individual self-care, the health professionals involved in the rehabilitation of burn patient should be informed about the risks of oral diseases and encourage them to refer the case; although this could be difficult because the current system protocol is deficient [47]. Additionally, the health system authorities could provide a friendly environment and atmosphere for the patients; for example, by having trained health professionals who understand their needs. However, a few issues are still not clear. It is a question whether the participants were dentally fit before the burn incidence and their status deteriorated because of it. It is also unclear if an oro-facial burn patient is referred to a dentist for a consultation immediately after the burn treatment completes. Studies on the relationship between oral health of burn patients and, oral health related quality of life and psychological status relating to facial appearance such as self-esteem and depression are on-going as part of the current investigation (drafts).

Originality is one of the strength of this study as it explored a niche oral health issue in a relatively small and less attended community. The study included a wide range of cases from the largest burn care centre that accepted patients from nationwide, hence may represent, to an extent, the oro-facial burn patients in general but limited to the Pakistani population. The results of this study should be interpreted with caution because of several limitations that may biased the results. The absence of information on the dental status before the burn event and cross-sectional study design limits the causal and temporal inference that the burn event is responsible for the outcomes in the patients. One of the challenging issues in this study is that much persuasion, assurance and patience are required to get the participation because the patients were psychologically and socially disturbed, hesitant, resistant, shy and afraid. It was difficult to obtain the correct and accurate information from the patients because these and, also due to pain as mentioned earlier and their educational background. Hence, the data collection process was one of the most difficult phases in the study as it was a complex, time consuming and exhausting procedure to be carried out while keeping the participants comfortable. Because of these and cost of travelling, the burn patient were not willing to and; for those who were given an appointment, did not; return for clinical reassessment, hence the reliability study was not carried out. Another potential bias is from the self-reported health behaviours and barriers to health care utilization where the participants had to recall events that occurred in the past year. For the barriers to utilization of health care, the use of the first response only in the analysis may underestimate the effect of the themes because a similar response that is repeated as a second response was not included.

## Conclusion

This study showed that patients with oro-facial burn injury have poor oral health status and that oral health behaviours, particularly the oral hygiene practice, dental visits and dental anxiety are the main modifiable factors that influence their oral health status. Referring the burn patients to a dentist at an early stage may prevent further deterioration of oral health, thus should be part of the post-emergency burn care.

## Data Availability

At present, the data related to the report is available upon reasonable request from the corresponding author because it is linked to a larger dataset of a PhD project which further reports are being prepared. It is the intention to make the whole dataset available on a platform such as the BMC Research Notes after the reports are completed.

## Abbreviations

CPI: Community Periodontal Index
DMFT: Decayed, Missing, Filled Teeth
OHI-S: Simplified Oral Hygiene Index
TBSA: Total body surface area
WHO: World Health Organization
